# Identification of specialized pro-resolving mediator clusters from healthy adults after intravenous low-dose endotoxin and omega-3 supplementation: a methodological validation

**DOI:** 10.1038/s41598-018-36679-4

**Published:** 2018-12-21

**Authors:** Paul C. Norris, Ann C. Skulas-Ray, Ian Riley, Chesney K. Richter, Penny M. Kris-Etherton, Gordon L. Jensen, Charles N. Serhan, Krishna Rao Maddipati

**Affiliations:** 1 Center for Experimental Therapeutics and Reperfusion Injury, Department of Anesthesiology, Perioperative and Pain Medicine and Brigham and Women’s Hospital and Harvard Medical School, Boston, MA 02115 USA; 2 Department of Nutritional Sciences, University of Arizona, Tucson, AZ 85721 USA; 3 Department of Nutritional Sciences, Pennsylvania State University, University Park, PA 16802 USA; 4 Larner College of Medicine, University of Vermont, Burlington, VT 05405 USA; 5 Department of Pathology, Wayne State University School of Medicine, Detroit, Michigan USA

**Keywords:** Innate immunity, Acute inflammation

## Abstract

Specialized pro-resolving mediator(s) (SPMs) are produced from the endogenous ω-3 polyunsaturated fatty acids (PUFA), eicosapentaenoic acid (EPA) and docosahexaenoic acid (DHA), and accelerate resolution of acute inflammation. We identified specific clusters of SPM in human plasma and serum using LC-MS/MS based lipid mediator (LM) metabololipidomics in two separate laboratories for inter-laboratory validation. The human plasma cluster consisted of resolvin (Rv)E1, RvD1, lipoxin (LX)B_4_, 18-HEPE, and 17-HDHA, and the human serum cluster consisted of RvE1, RvD1, AT-LXA_4_, 18-HEPE, and 17-HDHA. Human plasma and serum SPM clusters were increased after ω-3 supplementation (triglyceride dietary supplements or prescription ethyl esters) and low dose intravenous lipopolysaccharide (LPS) challenge. These results were corroborated by parallel determinations with the same coded samples in a second, separate laboratory using essentially identical metabololipidomic operational parameters. In these healthy subjects, two ω-3 supplementation protocols (Study A and Study B) temporally increased the SPM cluster throughout the endotoxin-challenge time course. Study A and Study B were randomized and Study B also had a crossover design with placebo and endotoxin challenge. Endotoxin challenge temporally regulated lipid mediator production in human serum, where pro-inflammatory eicosanoid (prostaglandins and thromboxane) concentrations peaked by 8 hours post-endotoxin and SPMs such as resolvins and lipoxins initially decreased by 2 h and were then elevated at 24 hours. In healthy adults given ω-3 supplementation, the plasma concentration of the SPM cluster (RvE1, RvD1, LXB_4_, 18-HEPE, and 17-HDHA) peaked at two hours post endotoxin challenge. These results from two separate laboratories with the same samples provide evidence for temporal production of specific pro-resolving mediators with ω-3 supplementation that together support the role of SPM *in vivo* in inflammation-resolution in humans.

## Introduction

Inflammation arises from responses to tissue injury and microbial stimuli to prevent the spread of infection^1^. Failure to resolve excessive inflammation is a central component of many chronic diseases^1–4^, including atherosclerosis and rheumatoid arthritis, thus contributing to the burden on public health. Nutritional intervention studies suggest that intake of docosahexaenoic acid (DHA), eicosapentaenoic acid (EPA), and other ω-3 polyunsaturated fatty acids (PUFA) may provide tissue protection^4^. The ω-3 PUFA are precursors for specialized pro-resolving mediators (SPM) that include resolvins, protectins and maresins that are produced in the resolution phase of acute inflammation. By definition, each SPM pathway stimulates resolution of inflammation and infections by limiting the exposure to pathogens and collateral damage from tissue-destructive neutrophils. SPMs enhance innate host defense responses that include macrophage phagocytosis of apoptotic neutrophils and microbes^3^. These proresolving mechanisms include limiting neutrophil infiltration and stimulation of macrophage mediated uptake of apoptotic neutrophils, cellular debris and microbes. Each action is stimulated at pico to nanomolar ranges of SPM, requiring stereospecific biosynthesis (reviewed in ref.^3^). Identification and profiling of SPMs has recently been operationalized with liquid chromatography-mass spectrometry (LC-MS) based approaches by multiple laboratories^5–7^. This has enabled elucidation of specific functional SPM clusters in several human tissues and fluids, including blood^5–7^, placenta^8^, and emotional tears^9^.

**Figure 1:**
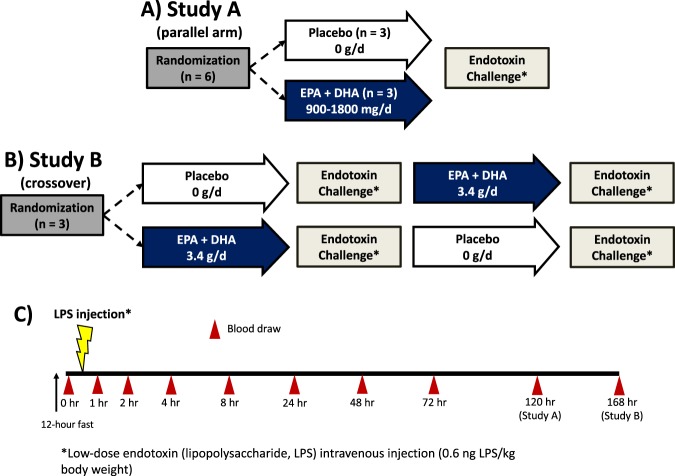
Study Design and Sampling Schematic. (**A**) Design of 5-month parallel arm supplementation study. (**B)** Design of 8-week supplementation crossover study. (**C**) Timing of blood sampling during the endotoxin (lipopolysaccharide, LPS) challenge testing visit; all time points were the same for both studies except for 120 hrs (measured in Study **A**) and 168 hrs (measured in Study **B**). Participants were required to fast for 12 hours prior to endotoxin administration.

In the present report, we demonstrate alignment between two independent laboratories for the identification and quantification of lipid mediators (LM) and SPM via metabololipidomic profiling of subjects who received intravenous low-dose endotoxin (lipopolysaccharide; LPS) and ω-3 PUFA supplementation in two studies (Fig. 1 and Table 1). In Study A, healthy volunteers were randomized to 900–1800 mg/d EPA and DHA (as the triglyceride form of a fish oil dietary supplement) or a soybean oil placebo for five months followed by intravenous low-dose endotoxin (lipopolysaccharide, LPS), which is a model of acute, systemic inflammatory challenge^10^. In Study B, which utilized a randomized crossover design, healthy men were given 3400 mg/d EPA or DHA and an olive oil placebo, each for 8 weeks prior to low-dose intravenous endotoxin challenge, with an 8-week washout (16 weeks separating each LPS testing visit). Using the above-specified metabololipidomic profiling approach with serum and plasma, we identified a cluster of potent bioactive^3–5^ pro-resolving mediators consisting of resolvin E1 (5*S*, 12*R*, 18*R*-trihydroxy-eicosa-6*Z*, 8*E*, 10*E*, 14*Z*, 16*E*-pentaenoic acid; RvE1), resolvin D1 (7*S*, 8*R*, 17*S*-trihydroxy-docosa-4*Z*, 9*E*, 11*E*, 13*Z*, 15*E*, 19*Z-*hexaenoic acid; RvD1), aspirin-triggered [R-epimer] (AT)-lipoxin A_4_ (5*S*, 6*R*, 15*S-*trihydroxy-eicosa-7*E*, 9*E*, 11*Z*, 13*E*-tetraenoic acid; LXA_4_), 18-hydroxy-eicosapentaenoic acid (HEPE), and 17-hydroxy-docosahexaenoic acid (HDHA) and elucidated their temporal regulation during inflammatory challenge and ω-3 PUFA supplementation.

**Table 1:** Participant characteristics^a^.

	Study A (n = 6)^b^		Study B (n = 3)^c^	
	Placebo (n = 3; 1M, 2F)	EPA and DHA (n = 3; 2M, 1F)	Placebo	EPA and DHA
Age (y)	24 ± 2 (21–28)	25 ± 0.3 (24–25)	29 ± 1.7 (29–32)	29 ± 1.7 (29–32)
Body mass index (kg/m^2^)	26.6 ± 0.7 (25.6–28.0)	23.8 ± 2.0 (21.4–27.7)	25.2 ± 2.1 (22.5–29.3)	25.9 ± 2.3 (24.5–30.4)
Systolic blood pressure	121 ± 4 (114–126)	118 ± 3 (114–124)	120 ± 3 (115–124)	119 ± 4 (115–127)
Diastolic blood pressure	77 ± 2 (74–80)	69 ± 6 (58–76)	77 ± 4 (70–85)	72 ± 2 (68–76)
TC (mg/dL)	178 ± 20 (157–218)	120 ± 10 (104–137)	136 ± 15 (111–163)	139 ± 14 (111–157)
LDL-C (mg/dL)	112 ± 15 (90–140)	58 ± 8 (52–67)	72 ± 12 (50–93)	77 ± 14 (52–102)
HDL-C (mg/dL)	46 ± 10 (27–56)	53 ± 6 (41–59)	43 ± 4 (41–50)	49 ± 6 (47–60)
TC:HDL-C	4.2 ± 0.9 (2.9–5.8)	2.3 ± 0.1 (2–2.5)	3.3 ± 0.6 (2.7–4.4)	3.0 ± 0.5 (2.4–4.0)
TG (mg/dL)	97 ± 15 (68–113)	45 ± 4 (41–53)	109 ± 31 (60–166)	66 ± 7 (57–80)
CRP (mg/L)	1.6 ± 0.7 (0.7–3.0)	0.3 ± 0.1 (0.2–0.6)	0.3 ± 0.1 (0.2–0.4	0.2 ± 0.1 (0.2–0.3)
Peak CRP (mg/L)	20.4 ± 1.1 (18.4–22.2)	17 ± 2.1 (14.1–21)	16.3 ± 2.2 (12–19)	14.2 ± 3.2 (9.6–20.2)
TNF-α (pg/mL)	1.6 ± 0.1 (1.3–1.7)	1.5 ± 0.1 (1.4–1.7)	1.0 ± 0.2 (0.7–1.3)	0.8 ± 0.1 (0.7–1.0)
IL-6 (pg/mL)	1.7 ± 0.6 (0.6–2.3)	1.0 ± 0.2 (0.8–1.3)	0.9 ± 0.2 (0.5–1.3)	0.8 ± 0.1 (0.6–1.1)
*Erythrocyte fatty acid content (% of total fatty acids)*				
Linoleic acid (LA; 18:2n6)	13.6 ± 1.04 (12.5–15.6)	12.5 ± 0.52 (11.6–13.4)	12.6 ± 0.29 (12.1–13.0)	12.1 ± 0.61 (11.3–13.3)
Arachidonic acid (AA; 20:4n6)	15.7 ± 0.60 (14.9–16.9)	13.8 ± 0.66 (12.8–15.0)	16.5 ± 0.97 (14.7–18.0)	14.4 ± 0.34 (13.9–15.0)
Eicosapentaenoic acid (EPA; 20:5n3)	0.44 ± 0.05 (0.39–0.55)	2.47 ± 0.71 (1.32–3.77)	0.31 ± 0.06 (0.28–0.37)	2.2 ± 0.09 (2.0–2.3)
Docosahexaenoic acid (DHA; 22:6n3)	3.81 ± 0.82 (2.67–5.39)	7.40 ± 0.80 (5.90–8.66)	3.80 ± 0.58 (3.20–4.94)	6.66 ± 0.16 (6.36–6.89)
Omega-3 index (EPA + DHA)	4.26 ± 0.87 (3.06–5.95)	9.88 ± 1.50 (7.23–12.4)	4.11 ± 0.60 (3.49–5.31)	8.85 ± 0.14 (8.58–9.06)

^a^Values represent participant characteristics directly prior to low-dose intravenous endotoxin administration and are presented as means ± SEM with ranges in parentheses.

^b^Parallel arm study in which participants received either placebo (0 mg/d EPA and DHA) or EPA and DHA supplementation for 5 months prior to endotoxin challenge. Participants in the EPA and DHA supplementation group received either 900 mg/d or 1800 mg/d.

^c^Crossover study in which participants received placebo and EPA and DHA (3.4 g/d) supplementation for 8 weeks prior to endotoxin challenge.

Abbreviations: CRP, C-reactive protein; HDL-C, high density lipoprotein-cholesterol; IL-6, interleukin-6; LDL-C, low density lipoprotein-cholesterol; TC, total cholesterol; TG, triglycerides; TNF-α, tumor necrosis factor-α.

## Results

**Figure 2:**
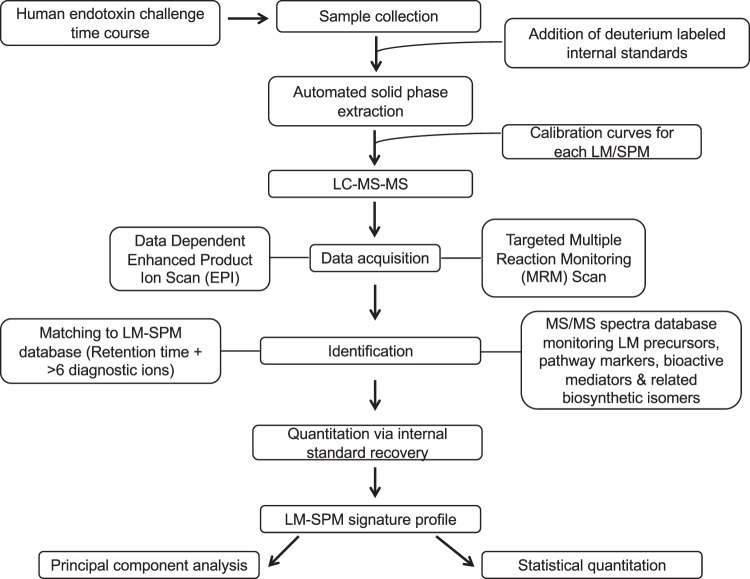
LM-SPM metabolomic protocol. Schematic of LM extraction, LC-MS/MS data acquisition and analysis implemented by two laboratories independently on same samples.

LM-SPM were profiled from serum in Study A and plasma in Study B using LC-MS/MS (Fig. 2, Supplementary Table 1, Supplementary Fig. 1). The duration and dose of ω-3 supplementation implemented in the two studies was selected based on results from earlier studies where supplementation reduced triglycerides and increased incorporation of both EPA and DHA into erythrocytes (i.e., ω-3 index^11,12^). Additional criteria for the individual studies were as follows: In Study A, supplementation was designed to reflect a range of dietarily achievable doses of EPA and DHA <2 grams/day, and the duration of supplementation was longer (~5 months) to permit incorporation of EPA and DHA into erythrocyte membranes^11^. In Study B, we implemented a crossover design with higher dosing of 3.4 g/d EPA and DHA and shorter duration (8–12 weeks) of supplementation (Fig. 1 illustration). Both studies resulted in increases in erythrocyte content of membrane EPA and DHA^11,12^ (and unpublished data below for Study B) and Table 1. Recently we found that coagulation activates production of a cluster of SPMs, including RvD1, resolvin D5 (7*S*, 17*S*-dihydroxy-docosa-4*Z*, 8*E*, 10*Z*, 13*Z*, 15*E*, 19*Z*-hexaenoic acid; RvD5), RvE1, maresin 1 (7*R*, 14*S*-dihydroxy-docosa-4*Z*, 8*E*, 10*E*, 12*Z*, 16*Z*, 19*Z*-hexaenoic; MaR1), and lipoxin B_4_ (5*S*, 14*R*, 15*S-*trihydroxy-eicosa-6*E*, 8*Z*, 10*E*, 12*E*-tetraenoic acid; LXB_4_)^13^. We therefore assessed the impact of omega-3 supplementation followed by endotoxin challenge on LM-SPM profiles obtained from serum (generated by coagulation *ex vivo*) as well as changes in plasma that reflect *in vivo* biosynthesis.

### Serum LM-SPM in Study A

**Figure 3:**
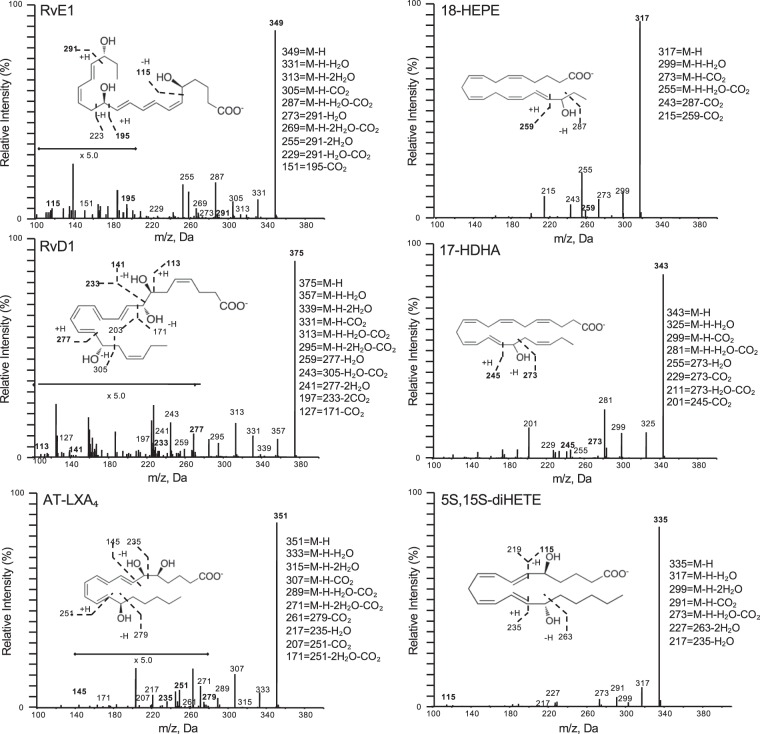
SPM identification with MS/MS fragmentation analysis in human serum. LC-MS/MS fragmentation and identification of SPMs and pathway markers based on presence of >6 diagnostic ions, denoted in the insets. Spectra are representatives from one of six healthy donors, and confirmation was carried out by both labs.

**Figure 4:**
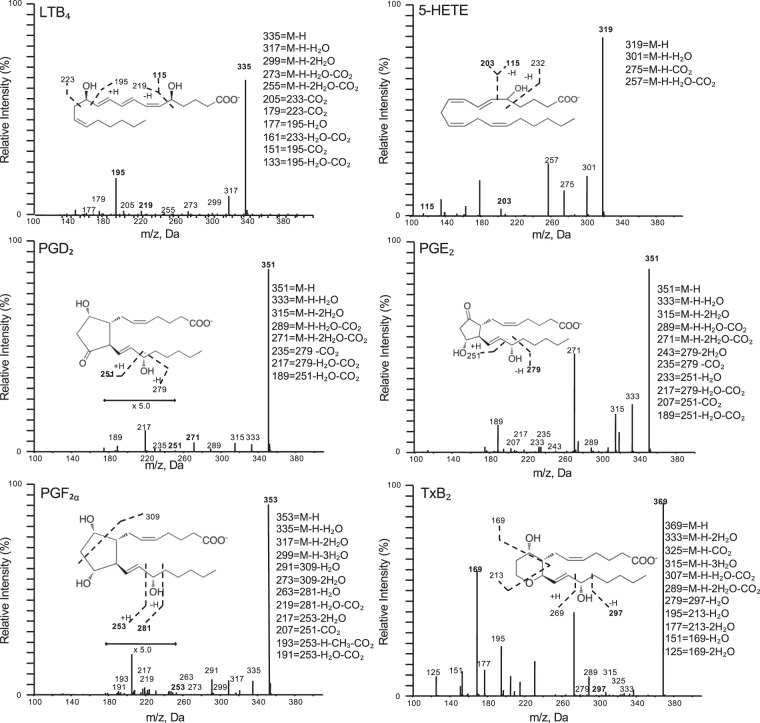
Pro-inflammatory leukotriene and prostaglandin identification with MS/MS fragmentation analysis in human serum. LC-MS/MS fragmentation and identification of prostaglandins, leukotrienes, and pathway markers based on presence of >6 diagnostic ions, denoted in the insets. Spectra are representatives from one of six healthy donors, and confirmation was carried out by both labs.

**Figure 5:**
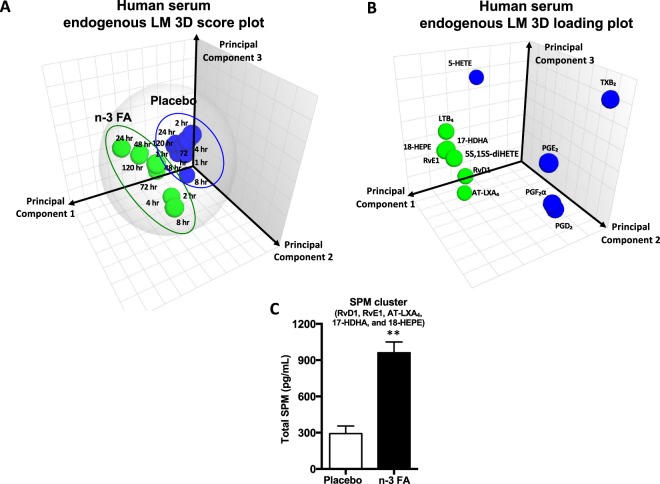
EPA and DHA supplementation increases SPM production in human serum after LPS challenge. Principal Component Analysis (PCA) for human LPS-challenged serum, with and without EPA + DHA supplementation. (**A**) 3-dimensional score plot of blood sampling time points; green circles are representative of ω-3 FA supplementation study group (mean of three donors), while blue circles are representative of placebo study group (mean of three donors). Gray ellipse denotes 95% confidence interval. (**B**) 3-dimensional loading plot of LM-SPMs identified in human serum upon LPS challenge; green circles are those mediators associated with the study group supplemented with ω-3 FA, while the blue circles are those mediators associated with the placebo study group. (**C**) Total SPM cluster and total prostaglandins, thromboxane, and LTB_4_ in human serum 120 h post LPS injection. Results are means ± SEM of 3 healthy subjects; **P < 0.01 for the ω-3 FA group vs. placebo group.

Using metabololipidomics focusing on cyclooxygenase and lipoxygenase pathways and products, we identified LM-SPM from each of the bioactive mediator metabolomes derived from DHA, EPA, and arachidonic acid (AA) (Supplementary Figs 2–5). In serum LM-SPMs from Study A: Both laboratories identified a cluster of SPMs in human serum that included RvE1, RvD1, AT-LXA_4_, 17-HDHA, and 18-HEPE (Figs 3 and 4; Supplementary Tables 2 and 3). Each of these mediators has potent pro-resolving and anti-inflammatory actions^3,14–16^ and were identified using MS-MS diagnostic ions as well as comparison with synthetic standards. All LM-SPMs and biosynthetic pathway markers were identified in accordance with published criteria^5,9^ that included matching retention time and at least six characteristic and diagnostic fragment ions. Principal component analysis indicated that serum SPMs associated with ω-3 supplementation as well as prostaglandins and thromboxane, which associated with placebo throughout the endotoxin challenge time course (Fig. 5A,B). Total amounts of the SPM cluster consisting of RvD1, RvE1, AT-LXA_4_, 17-HDHA, and 18-HEPE increased 229% in human subjects given ω-3 vs. subjects with placebo (Fig. 5C) at 120 hours post endotoxin challenge. To establish independent alignment in these results, a second aliquot of these coded samples was analyzed with metabololipidomics (operated and optimized as in Fig. 2) via a second laboratory. This second analysis identified the same SPM cluster, which was increased 169% in subjects receiving ω-3 supplementation vs. subjects with placebos.

**Figure 6:**
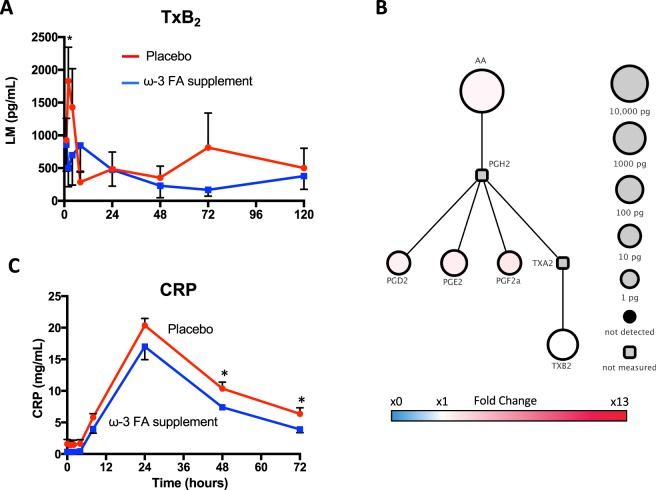
Time course of prostaglandins and thromboxane in human serum with LPS challenge. (**A**) Thromboxane concentrations during 120 h LPS time course in serum. (**B**) Prostaglandin and thromboxane corresponding pathway fold-change in ω-3 FA group vs placebo group at 0 h; circle size represents ω-3 FA group LM quantities (pg/mL) at 0 h. (**C**) C-reactive protein (CRP) concentrations during 120 h LPS time course in serum. Results are means ± SEM of 3 healthy subjects.

**Figure 7:**
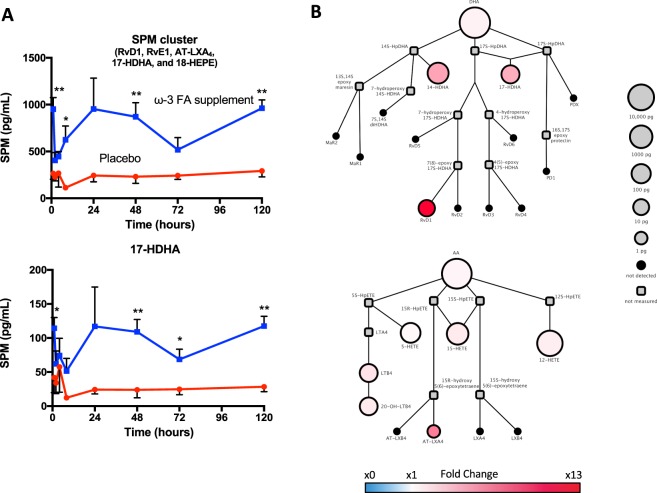
Time course of SPM in human serum with LPS challenge. (**A**) Total SPM cluster amounts (top) and 17-HDHA (bottom) during 120 h LPS time course in serum. (**B**) Corresponding DHA-derived pathway changes (top) and AA-derived SPM and leukotriene pathway changes between ω-3 FA group vs. placebo group at 0 h; circle size represents ω-3 FA group LM quantities in pg/mL at 0 h. Results are means ± SEM of 3 healthy subjects.

Time course analysis indicated that serum prostaglandin and thromboxane concentrations in serum were highest at early time points, 0–8 hours post endotoxin challenge (Fig. 6A,B and Supplementary Tables 2 and 3). In this subset of study participants (n = 3/group), TXB_2_ peaked at 2 hours with placebo and was statistically significantly reduced with ω-3 supplementation (Fig. 6A). C-reactive protein (CRP) concomitantly peaked at 24 hours and was significantly reduced in the ω-3 group vs. placebo group (Fig. 6C) at 48 and 72 h. A cluster of SPMs (RvD1, RvE1, AT-LXA_4_, 17-HDHA, and 18-HEPE) was increased in the serum of subjects taking ω-3 PUFA vs. placebo throughout the time course of endotoxin challenge (Fig. 7A–C and Supplementary Tables 2 and 3). This SPM cluster in the ω-3 group initially decreased at early time points (2 h) and increased at 24 hours post endotoxin challenge (Fig. 7A). These results demonstrate that ω-3 PUFA supplementation increases a cluster of specific pro-resolving mediators, namely RvD1, RvE1, AT-LXA_4_, 17-HDHA, and 18-HEPE in human serum. Thus, ω-3 PUFA supplementation increased the potential for blood cells to produce SPMs after endotoxin challenge, assuming that leukocytes and platelets are the main source of SPM in serum.

### Plasma LM-SPMs in Study B

**Figure 8:**
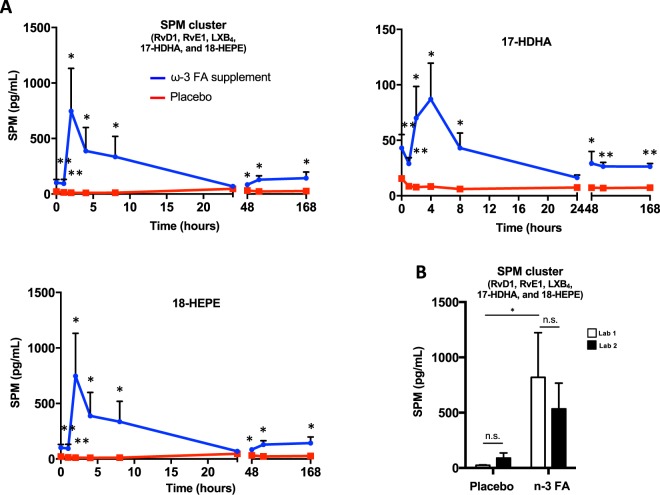
SPM production is increased in human plasma via ω-3 PUFA supplementation and immunologic challenge. (**A**) Total SPM cluster concentrations in human plasma with ω-3 PUFA supplementation or placebo 1–168 h post intravenous LPS injection; *P < 0.05, **P < 0.01 for ω-3 PUFA supplement group vs. placebo group with one-tailed ratio t-test. (**B**) Comparison of total SPM cluster concentrations in human plasma at 2 h between separate laboratories. Results are means ± SEM of 3 healthy subjects; *P < 0.05; ns = not statistically different using paired one-tailed ratio t-test.

To assess direct changes in human peripheral blood LM (prostaglandins and leukotrienes) and SPM with ω-3 supplementation as well as during endotoxin challenge, LM-SPM metabololipidomic profiling was carried out with human plasma from participants taking 4 grams/day of ω-3 ethyl esters (3.4 grams/day EPA and DHA ethyl esters) for 8–12 weeks followed by low dose intravenous LPS administration. Plasma was also obtained from the same participants after 8–12 weeks of placebo (olive oil) supplementation and a low dose intravenous LPS challenge. Treatments were received in random order with an 8-week washout period (crossover) between each supplementation period, resulting in at least 16 weeks between testing visits. In these plasma samples, RvD1, RvE1, LXB_4_, 17-HDHA, and 18-HEPE were identified (Fig. 8A and Supplementary Tables 4 and 5). This LM-SPM cluster was increased with ω-3 supplementation vs. placebo throughout the LPS time course (Fig. 8A). LM-SPM amounts peaked at 2 h post-LPS administration with ω-3 supplementation while such temporal changes were not observed with placebo (Fig. 8A). LM-SPM metabololipidomic analysis by the second laboratory identified these same mediators and additionally identified resolvin D2 (7*S*, 16*R*, 17*S*-trihydroxy-docosa-4*Z*, 8*E*, 10*Z*, 12*E*, 14*E*, 19*Z-*hexaenoic acid; RvD2), resolvin D4 (4*S*, 5*R*, 17*S*-trihydroxy-docosa-6*E*, 8*E*, 10*Z*, 13*Z*, 15*E*, 19*Z* hexaenoic acid; RvD4), RvD5, and RvD6. Both laboratories quantified consistent increases in SPM cluster amounts with ω-3 supplementation vs. placebo and repeat measurements of total SPM cluster amounts were not statistically significantly different between the two labs (Fig. 8B). These results demonstrate that a specific SPM cluster, namely RvD1, RvE1, LXB_4_, 17-HDHA, 18-HEPE, is increased in plasma in response to endotoxin challenge in individuals with ω-3.

In the present study, distinct LM-SPM were quantifiable in a volume of 1 mL of human plasma or serum with ω-3 supplementation and endotoxin challenge (Figs 3 and 4, Supplementary Tables 2–5), even in the presence of substantial matrix suppression associated with these biological samples (Supplementary Fig. 1) and the limits of detection with the workup and instruments used. Specifically, these include RvD1, RvE1, AT-LXA_4_, LXB_4_, 17-HDHA, and 18-HEPE. Both 17-HDHA and 18-HEPE, which are bioactive SPM^14,15,17^ as well as precursors to resolvins, were consistently identified in quantifiable concentrations by both laboratories in the present study, and are thus strong candidates for plasma and serum clinical biomarkers of SPM and resolution of inflammation. RvD1 was identified (Supplementary Tables 4 and 5) in human plasma following endotoxin challenge, while other D-series resolvins (RvD2 – RvD6) that are present in human blisters^18^, lymph^5^ and breast milk^19,20^ were not quantifiable in these blood specimens from healthy individuals (Supplementary Tables 2 and 3). Thus, to optimize for future studies, ideally >1 mL plasma and serum from healthy subjects would improve identification of LM-SPM. Since SPMs including those identified in the serum and plasma clusters in the present study are increased via coagulation^13^, a dose response of lipid mediator production between the two (i.e. plasma vs. serum) could not be evaluated using the two study protocols and will require further investigations.

Interlaboratory results acquired in the present study were in agreement in the identification of clusters of SPMs in human serum and plasma that increased following ω-3 supplementation. Both laboratories employed essentially identical instrumentation and data acquisition parameters, albeit non-statistically significant differences in quantification between the two labs (Fig. 8B) appear to reflect differences in sample extraction and data processing methods. Hence, it is imperative that standardization of LC-MS/MS metabololipidomic platforms across separate laboratories is validated for further improvement of quantitation of LM and SPM. Variations in identification and quantification of specific lipids by many separate laboratories in the field^21^ likely reflect lab-dependent sample workup including lipid mediator solid-phase extraction procedures, liquid chromatography solvent systems and lack of deuterium-labeled internal standards for quantitation, as well as instrument specifications with respect to mass spectrometry collision energy and ionization parameters.

## Discussion

In the present study, we provide evidence for the *in vivo* production of SPMs in humans with ω-3 supplementation and endotoxin challenge. These results are consistent with the many animal and human studies that have utilized LC-MS/MS analysis for SPM identification and quantification. Along these lines, in animal models of disease and inflammation, resolvins and other members of the SPM superfamily are identified and present at concentrations that are biologically active (reviewed in refs^3,9,13^). For example, increasing ω-3 PUFA via transgenic overexpression of *fat-1* in mice enhances the formation of RvE1, resolvin D3 (4*S*, 11*R*, 17*S*- trihydroxy-docosa-5*Z*, 7*E*, 9*E*, 13*Z*, 15*E*, 19*Z* hexaenoic acid; RvD3), and protectin D1 (10*R*, 17*S*-dihydroxy-docosa-4*Z*, 7*Z*, 11*E*, 13*E*, 15*Z*, 19*Z*-hexaenoic acid, PD1; also known as neuroprotectin D1 [NPD1]), reduces inflammation, and enhances protection against tissue injury in colitis^22^. During intestinal ischemia/reperfusion in mice, production of LXA_4_ and 18-HEPE is increased and blockade of LXA_4_ signaling to its receptor (ALX) mitigates its anti-inflammatory and pro-resolving actions^23,24^. In sterile murine peritonitis, RvD1, RvD2, RvD5, PD1, MaR1, and LXA_4_ are produced during the resolution phase^25^, whereas, in infectious murine peritonitis with pathogenic *E. coli*, RvD1, RvD5, and PD1 are produced and lower antibiotic requirements for bacterial clearance^26^. In non-human primates, baboons infected with *S. pneumoniae* display diminished plasma levels of lipoxins and E-series resolvins, which are increased with therapeutic low doses of carbon monoxide^27^. In chronic inflammatory disease models, RvD1, RvD2, RvD3, and RvD4 are present in self-resolving murine arthritis, while RvD3 is reduced in delayed-resolving arthritis and reduces paw joint clinical scores, leukocytes, eicosanoids, and edema^28^. Additionally, AT-RvD1 and its precursor, 17R-HDHA, reduce inflammatory pain in an adjuvant-induced arthritis model^29,30^. Recently, in a murine model of Alzheimer’s disease, AT-LXA_4_ and RvE1 were endogenously produced via sphingosine kinase 1 (SphK1)-dependent acetylation of cyclooxygenase (COX)-2 and reduced disease pathology via enhancement of microglial phagocytosis^31^. Thus, the identification of 17R and 15S epimers of resolvins and lipoxins (triggered by aspirin or statins) in humans and animals can also be attributed to endogenous mechanisms of epimer biosynthesis in addition to those triggered by drugs (i.e. 17R-, 18R-, and 15R-SPM epimers)^3^.

In humans, both individual SPMs and their clusters are present in biologically active concentrations in inflammatory exudates and physiologic tissues and fluids as determined by targeted LC-MS/MS based approaches. In human peripheral blood, multiple independent laboratories collectively identified SPMs in plasma (first with identification and complete stereochemical assignment of RvE1 via MS)^3,32^, as well as a plasma SPM cluster consisting of RvE1, resolvin E2 (5*S*, 18*R*-dihydroxy-eicosa-6*E*, 8*Z*, 11*Z*, 14*Z*, 16*E*-pentaenoic acid; RvE2), RvD1, 17-epi-RvD1, RvD2, RvD5, RvD6, PD1, 17-HDHA and 18-HEPE^5,6^, and a serum cluster consisting of RvD1, 17-epi-RvD1, RvD2, RvD3, PD1, MaR1, RvE1, RvE2, LXA_4_, LXB_4_, 17-HDHA, and 18-HEPE^5,6^. Recovery from strenuous exercise in healthy people coincides with increases in serum SPMs (RvD1, RvE1, LXA_4_, and LXB_4_), and this response is blocked by pretreatment with an oral NSAID, ibuprofen^33^. In patients with chronic daily headaches, a dietary intervention that increases ω-3 PUFA and reduces n-6 PUFA increases plasma 17-HDHA, 18-HEPE and resolvin D2 with concomitant reduction in headache pain^34^. Following sepsis, plasma concentrations of RvE1, RvD5, and 17-epi-PD1 significantly increase in human sepsis non-survivors vs. surviving sepsis subjects and are potential biomarkers for critical illness^35^.

At birth, SPMs are present in human umbilical cord blood (RvE1, RvE2, resolvin E3 (5*S*, 18*R*-dihydroxy-eicosa-6*E*, 8*Z*, 11*Z*, 14*Z*, 16*E*-pentaenoic acid; RvE3), RvD1, 17-epi-RvD1, RvD2, 17-HDHA, and 18-HEPE)^36,37^ and placenta (RvD1, 17-epi-RvD1, RvD2, PD1, 17-HDHA, and 18-HEPE)^8^. Prenatal ω-3 PUFA supplementation increases 18-HEPE and 17-HDHA concentrations in human maternal and cord blood^7,36^, as well as in human placenta^7^, which may support early immune functions in utero and in newborns. Along these lines, human breast milk also contains a bioactive cluster of SPMs that consists of RvD1, RvD2, RvD3, 17-epi-RvD3, RvD4, PD1, MaR1, RvE1, RvE2, RvE3, LXA_4_, LXB_4_, 17-HDHA, and 18-HEPE^20,28,38,39^. Specific SPMs are also present in human lymphoid organs, which include human spleen SPMs consisting of RvD5, PD1, MaR1, RvE1, RvE2, RvE3, and LXA_4_ ^5^, and human axillary lymph node SPMs consisting of RvD1, RvD5, RvD6, RvE3, LXA_4_, and LXB_4_ ^5^.

A growing body of evidence from LM pathway metabololipidomics profiling indicates that SPM production is altered and often diminished in affected tissues and in circulation across a spectrum of human chronic inflammatory diseases. In human synovial fluid from rheumatoid arthritis patients, RvD1, 17-epi-RvD1, RvD2, RvD3, RvE1, RvE2, RvE3, PD1, MaR1, 17-HDHA, and 18-HEPE are present^19,40,41^, and RvD3 is reduced in serum from rheumatoid arthritis patients^19^. RvD1 is significantly reduced in the vulnerable regions of human atherosclerotic plaques^42^, and in omental adipose tissue from obese patients, D-series resolvins, E-series resolvins, PD1, MaR1, and lipoxins are reduced relative to concentrations of leukotriene B_4_ (5*S*, 12*R*-dihydroxy-eicosa-6*Z*, 8*E*, 10*E*, 14*Z*-tetraenoic acid; LTB_4_) and prostaglandins^43^. In brain and cerebrospinal fluid from patients with Alzheimer’s disease, RvD1 and LXA_4_ are decreased. SPMs have also been identified in human biological fluids and exudates, thus providing minimally invasive pools from which to measure human LM-SPM profiles that may reflect disease and nutritional status. For example, in human emotional tears, RvD1, RvD2, RvD5, PD1, LXA_4_ and AT-LXA_4_ are identified and are reduced overall in female donors vs. male donors^9^. In cantharidin-induced human blisters, RvD1 and RvD2 are identified and are increased in female subjects vs. male subjects^18^. Specific SPMs are also identified in human urine, namely RvD1, 17-epi-RvD1, and RvE2, from smokers and non-smokers^44^.

SPMs exert potent pro-resolving and anti-inflammatory actions (at pM-nM range) on human leukocytes^3^. RvD1, RvD2, and LXA_4_, each at 1 nM concentration, stimulate shape change and stop neutrophil chemotaxis toward IL-8 gradients^41^. By definition, resolvins, protectins, maresins, and lipoxins are pro-resolving mediators because they reduce pro-inflammatory stimuli and stimulate human macrophage efferocytosis of apoptotic neutrophils and microbial clearance^13,45–48^. In the present experiments, we have obtained evidence for increased SPMs in humans following ω-3 PUFA supplementation, which supports the theory that they are produced in human tissue at concentrations that are biologically active. These concentrations (at or above 100 pM) are produced in human peripheral blood and affect the functions of both neutrophils and monocytes (at the single cell level as determined by CyTOF mass cytometry), as well as increasing phagocytosis and killing of pathogenic *E. coli*^13^. Biomarker concentrations of ω-3 PUFA are associated with reduced incidence of fatal coronary heart disease^4,49,50^, and it has recently been established that ω-3 PUFA supplementation at doses up to 10 g/day (EPA and DHA) does not increase the risk of bleeding or affect other clinically meaningful coagulation parameters^51^. It is also noteworthy that both 17-HDHA and 18-HEPE are biosynthetic intermediates in human leukocyte SPM production^3^, and each is also reported to carry potent bioactions of their own. Namely, 18-HEPE displays vascular actions^15^ and 17-HDHA has demonstrated potent reduction of arthritic pain^17^ and reduces viral H1N1 infections by enhancing antibody mediated immune responses^14^.

Local organ production of SPM, e.g. in human breast milk^28^, tears^9^, and muscle tissue^33^, may be the source of some blood plasma SPMs. On the other hand, local inactivation of SPM may contribute to the absence or diminished levels of select SPM in plasma. For example, at sites of inflammation, as in inflammatory exudates, leukocytes can convert SPM to further metabolites that carry diminished bioactivity via dehydrogenation and omega-oxidation, e.g. 15-oxo-LXA_4_, 13,14-dihydro-LXA_4_, 13,14-dihydro-15-oxo-LXA_4_ ^52^, 22-hydroxy-PD1^53^, 17-oxo-RvD1^54^, 18-oxo-RvE1^55^, 14-oxo-MaR1 and 22-OH-MaR1^56^. For each of the SPMs that undergo rapid, local enzymatic conversion and inactivation, specific mimetic analogs have been introduced as potential therapeutic agonists of resolution^3,57^. Thus, the potential physiologic significance of circulating SPM remains of interest.

In the present report with LPS challenge of the subject, we identified specific clusters of both LM (PG and LT) and SPMs in human plasma (RvE1, RvD1, LXB_4_, 17-HDHA, and 18-HEPE) and serum (RvE1, RvD1, AT-LXA_4_, 17-HDHA, and 18-HEPE) that were specifically increased with ω-3 PUFA supplementation. Differences in composition between serum and plasma SPM clusters indicated the presence of AT-LXA_4_ in serum with LXB_4_ in plasma. The presence of RvE1, RvD1, and LXB_4_ in plasma may be the result of LPS activation of platelets and leukocytes since these mediators can also be produced via platelet-leukocyte transcellular biosynthesis during coagulation^13^. For example, transcellular biosynthesis with human platelets and leukocytes produces lipoxins^58^. Serum formation during coagulation in the present study was preceded by endotoxin challenge, which might alter the SPM cluster profile, so these may reflect increases in further metabolism of LXB_4_ (e.g., either 20-OH-LXB_4_ or dehydrogenation to 5-oxo-LXB_4_) to products not targeted in the present approach.

In the case of AT-LXA_4_, this natural lipoxin epimer (15R-LXA_4_), in addition to its biosynthesis via aspirin acetylation of COX-2, is also biosynthesized via COX-2 nitrosylation^59,60^ and/or via the recently discovered SphK1-dependent COX-2 acetylation which increases biosynthesis of R containing epimers of lipoxins and resolvins^31^. Thus, the appearance of AT-LXA_4_ in serum as potential other AT-epimers of SPM could reflect aspirin ingestion or any of these endogenous mechanisms (note: subjects abstained from taking aspirin per study protocol). These results require further investigation to assess changes in specificity of LM production and metabolism following LPS challenge and coagulation.

The origins of plasma resolvins, lipoxins, 17-HDHA, and 18-HEPE remain to be identified. It is possible that they originate in organs that produce SPM such as bone marrow, adipose tissue, or sites of local coagulation *in vivo* that then appear in plasma. These local sites of LM biosynthesis and the potential physiologic functions of plasma SPM warrant further study. Nonetheless, the present results provide evidence for potential counter-regulation of inflammation in human circulation and host defense^13^ via endogenous production of SPMs in humans with ω-3 FA supplementation consistent with their pro-resolving actions in experimental animal systems^3^.

Although the number of healthy human subjects in the current study was small, these results were cross validated between two separate laboratories using blinded, coded samples and indicate that it is possible to align LC-MS/MS identification and profiling of LM-SPMs between different laboratory settings for larger studies. By optimization of instrument parameters in separate laboratories, sample preparation and rigorous workup procedures, this LM-SPM profiling approach is useful for directly assessing the impact of drugs, nutrition and disease phenotypes in these mediator pathways and potent bioactive products such as the resolvins and other SPMs. Our results provide cross validation and identification of SPM as well as evidence for temporal production of specific pro-resolving mediators with ω-3 PUFA supplementation. These results support an immunoresolvent role of SPM in inflammation resolution in humans challenged with endotoxin that now warrants further investigations with other natural host responses.

## Materials and Methods

### Human ω-3 PUFA supplementation and low-dose endotoxin challenge

Both studies A and B were conducted and annually approved by the Pennsylvania State University Institutional Review Board, and all participants provided written informed consent. All experiments were performed in accordance with relevant guidelines and regulations. Study A was approved by the FDA and registered at clinicaltrials.gov with the number NCT01078909. Study B was approved by the FDA and registered at clinicaltrials.gov with the number NCT01813110.

In study A, healthy volunteers (n = 6) were randomly assigned to placebo capsules containing 0 mg EPA and DHA (n = 3) or to ω-3 supplementation (n = 3; EPA and DHA esterified in the triglyceride form; Nordic Naturals). Participants in the ω-3 supplementation group received either 900 mg/d with 550 mg as EPA and 350 mg as DHA (n = 1) or 1800 mg/d with 1100 mg as EPA and 700 mg as DHA (n = 2) for 5 months prior to low-dose intravenous endotoxin injection (0.6 ng LPS/kg body weight). Participants were required to fast for 12 hours prior to LPS administration. Blood samples were obtained at 9 different time points (0 hr, 1, 2, 4, 8, 24, 48, 72, and 120 hours post LPS administration; see Fig. 1 illustration).

In Study B, healthy men (n = 3) were supplemented for 8 weeks with 3.4 grams/day EPA and DHA (in the form of four capsules each containing 460 mg of EPA-ethyl ester and 380 mg of DHA-ethyl ester; supplied by Pronova BioPharma) and olive oil for placebo that did not contain either EPA or DHA, in random order, with an 8-week washout period in between supplementation periods. The low-dose endotoxin challenge procedure was the same as that used in Study A, except that the 120-hour blood draw was replaced by a blood sample obtained at 168 hours post LPS administration.

### Lipid mediator metabololipidomics

Human plasma or serum samples were analyzed by two independent labs (Boston, MA and Detroit, MI) following similar protocols and shared internal standards. In both labs, the samples (approximately 1 mL) were thawed on ice and supplemented with 500 pg each of deuterated (d)8-5-hydroxy-eicosatetraenoic acid (HETE), d5-RvD_2_, d5-LXA_4_, d4-LTB_4_, d4-prostaglandin E_2_ (9-oxo-11α, 15*S*-dihydroxy-prosta-5*Z*, 13*E*-dien-1-oic acid; PGE_2_) (Cayman Chemical Company) in methanol before further processing. From here on, the two labs followed their own procedures for extraction and LC-MS analysis. In lab 1 (Boston, MA), four volumes of ice-cold LC-MS grade methanol was added to each sample and placed on ice for 45 minutes in the dark to allow for protein precipitation, followed by a centrifugation step (3,000 rpm, 10 min, 4 °C). Supernatants were collected from each sample, and solid phase extraction was carried out according to optimized and reported methods^9^. Methyl formate fractions were then analyzed by liquid chromatography-tandem mass spectrometry system, Qtrap 5500 (AB Sciex) equipped with a Shimadzu LC-20AD HPLC (Tokyo, Japan). The column implemented on this system was a Poroshell 120 EC-18 column (100 mm × 4.6 mm × 2.7 μm; Agilent Technologies, Santa Clara, CA, USA), housed in a column oven regulated at 50 °C, and lipid mediators (LMs) were eluted in a gradient of methanol/water/acetic acid from 55:45:0.01 (v/v/v) to 98:2:0.01 at 0.5 mL/min flow rate. Targeted multiple reaction monitoring (MRM) and EPI were utilized in order to quantify the mediator levels, with MS/MS matching to at least 6 diagnostic and signature ion fragments per molecule. A final analytic quantitation and recovery was performed using the deuterium labeled internal standards, and a LM-SPM profile was produced for each donor. All materials and methods, beginning with sample preparation and finishing with a LM-SPM profile, were completed by two independent labs simultaneously at separate locations.

In lab 2, (Detroit, MI), LC-MS grade methanol was added to the internal standard supplemented samples to a final concentration of 15%. The samples were sonicated in a bath sonicator for 2 min and left on ice for 1 h in dark. The samples were applied to pre-conditioned C18 solid phase extraction cartridges (StrataX C18, 30 mg, Phenomenex, conditioned with 2 ml methanol followed by 2 ml water containing 15% methanol), washed with 2 ml 15% methanol in water followed by 2 ml hexane, and dried under vacuum. The cartridges were eluted directly into HPLC autosampler vials with 1 ml methanol containing 0.1% formic acid. The eluates were evaporated to dryness under a gentle stream of nitrogen while maintaining the external temperature at 25 °C. The dried residue was immediately reconstituted in methanol, vials flushed with nitrogen, capped, and stored at −80 °C until analysis. At the time of LC-MS analysis, the samples were thawed to room temperature, and equal volume of 25 mM aqueous ammonium acetate was added, vortex mixed, and loaded in the autosampler maintained at 15 °C. HPLC is performed on a Prominence XR system (Shimadzu) using Luna C18 (3 µ, 2.1 × 150 mm) column. The mobile phase consists of a gradient between A: methanol-water-acetonitrile (10:85:5 v/v) and B: methanol-water-acetonitrile (90:5:5 v/v), both containing 0.1% ammonium acetate. The gradient program with respect to the composition of B is as follows: 0–1 min, 50%; 1–8 min, 50–80%; 8–15 min, 80–95%; and 15–17 min, 95%. The flow rate is 0.2 ml/min. The HPLC eluate is directly introduced to ESI source of QTRAP5500 mass analyzer (SCIEX) in the negative ion mode with the following conditions: Curtain gas, GS1, and GS2: 35 psi, Temperature: 600 °C, Ion Spray Voltage: −2500 V, Collision gas: low, Declustering Potential: −60 V, and Entrance Potential: −7 V. The eluate is monitored by Multiple Reaction Monitoring (MRM) method to detect unique molecular ion – daughter ion combinations for each of the transitions listed in Supplementary Data, Table 1. The MRM is scheduled to monitor each transition for 120 s around the established retention time for each lipid mediator. Optimized Collisional Energies (18–35 eV) and Collision Cell Exit Potentials (7–10 V) are used for each MRM transition. Mass spectra for each detected lipid mediator were recorded using the Enhanced Product Ion (EPI) feature to verify the identity of the detected peak in addition to MRM transition and retention time match with the standard. The data are collected using Analyst 1.6.2 software and the MRM transition chromatograms are quantitated by MultiQuant software (both from SCIEX). The internal standard signals in each chromatogram are used for normalization for recovery as well as relative quantitation of each analyte. For complete chemical names of lipid mediators in the current study, see ref.^9^.

### Statistical analysis

Groups were compared with Student’s t-test (two groups) using Prism version 6 (GraphPad, La Jolla, CA USA). The criterion for statistical significance was p < 0.05. Principal component analysis (PCA) was performed using SIMCA 13.0.3 software (MKS Data Analytics Solutions, Umeå, Sweden).

## Electronic supplementary material

Supplementary Figure 1 and Supplementary Tables 1-5

## Data Availability

LC-MS/MS data for lipid mediator retention time and fragmentation matching are available at: http://serhanlab.bwh.harvard.edu.
