# Author Correction: Identification of specialized pro-resolving mediator clusters from healthy adults after intravenous low-dose endotoxin and omega-3 supplementation: a methodological validation

**DOI:** 10.1038/s41598-019-56282-5

**Published:** 2019-12-19

**Authors:** Paul C. Norris, Ann C. Skulas-Ray, Ian Riley, Chesney K. Richter, Penny M. Kris-Etherton, Gordon L. Jensen, Charles N. Serhan, Krishna Rao Maddipati

**Affiliations:** 1 Center for Experimental Therapeutics and Reperfusion Injury, Department of Anesthesiology, Perioperative and Pain Medicine and Brigham and Women’s Hospital and Harvard Medical School, Boston, MA 02115 USA; 2 Department of Nutritional Sciences, University of Arizona, Tucson, AZ 85721 USA; 3 Department of Nutritional Sciences, Pennsylvania State University, University Park, PA 16802 USA; 4 Larner College of Medicine, University of Vermont, Burlington, VT 05405 USA; 5 Department of Pathology, Wayne State University School of Medicine, Detroit, Michigan USA

Correction to: *Scientific Reports* 10.1038/s41598-018-36679-4, published online 21 December 2018

This Article contains an error in Figure 8 where the total SPM graph is a duplication of the 18-HEPE graph, which has resulted in the panels for the SPM cluster and 18-HEPE to inadvertently appear to be very similar. The correct Figure 8 appears below as Figure 1.

**Figure 1:**
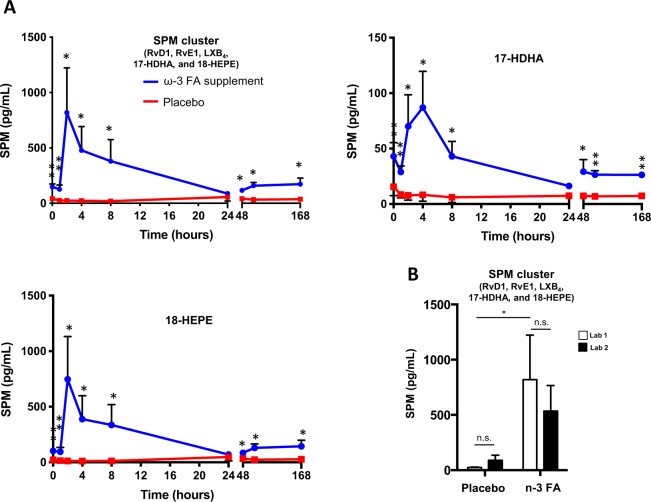
.

