# Supplementary material for: Identification of specialized pro-resolving mediator clusters from healthy adults after intravenous low-dose endotoxin and omega-3 supplementation: a methodological validation
Source: Sci Rep. 2018 Dec 21;8:18050. doi: 10.1038/s41598-018-36679-4 (PMC6303400; doi:10.1038/s41598-018-36679-4)
Supplement: Supplementary file 1 — Supplementary Figure 1 and Supplementary Tables 1-5 [file 41598_2018_36679_MOESM1_ESM.pdf]

Supplementary Information

Identification of specialized pro-resolving mediator clusters from healthy adults after intravenous low-dose endotoxin and omega-3 supplementation: a methodological validation

Paul C. Norris, Ann C. Skulas-Ray, Ian Riley, Chesney K. Richter, Penny M. Kris-Etherton, Gordon L. Jensen, Charles N. Serhan, and Krishna Rao Maddipati

Supplementary Figure 1

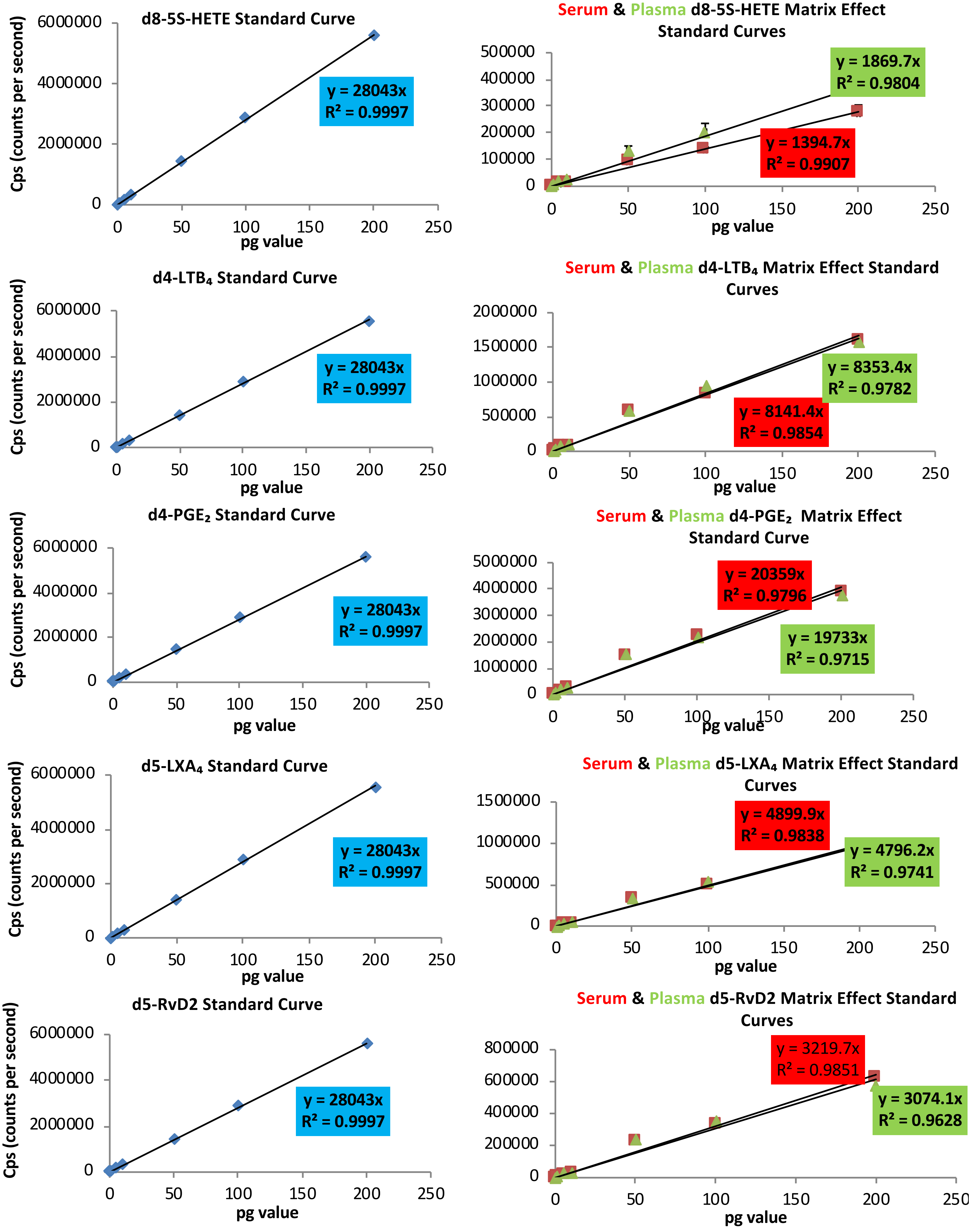

Supplementary Figure 1. Deuterium-labeled internal standard calibration curves, utilized for purposes of quantitative recovery. Deuterium-labeled standards were diluted in matrix obtained from 1.0 mL fresh human serum or plasma after solid-phase extraction and taken to LC-MS/MS.

Supplementary Table 1. LM-SPM parent and daughter ion fragmentation masses

**DHA bioactive metabolome**	**Q1**	**Q3**
RvD1	375	121
AT-RvD1	375	121
RvD2	375	215
RvD3	375	147
AT-RvD3	375	147
RvD5	359	199
RvD6	359	159
PD1	359	153
AT-PD1	359	153
10S,17S-diHDHA	359	153
22-OH-PD1	375	153
22-COOH-PD1	389	153
MaR1	359	221
7S,14S-diHDHA	359	250
4S,14S-diHDHA	359	101
**EPA bioactive metabolome**		
RvE1	349	195
RvE2	333	213
RvE3	333	201
**AA bioacive metabolome**		
LXA_4	351	115
AT-LXA_4	351	115
LXB_4	351	221
AT-LXB_4	351	221
5S,15S-diHETE	335	235
LTB_4	335	195
20-OH-LTB4		
20-COOH-LTB_4	365	195
5S,12S-diHETE	335	195
PGD_2	351	189
PGE_2	351	189
$PGF_{2\alpha}$	351	193
TXB_2	369	169
**DHA pathway markers**		
17-HDHA	343	245
14-HDHA	343	205
7-HDHA	343	141
4-HDHA	343	101
**EPA pathway markers**		
18-HEPE	317	259
15-HEPE	317	219
12-HEPE	317	179
5-HEPE	317	115
**AA pathway markers**		
15-HETE	319	259
12-HETE	319	179
5-HETE	319	115

Supplementary Table 2

Human serum LM-SPM profile from endotoxin challenge timecourse after placebo

Mediator		0 hour			1 hour			2 hours			4 hours			8 hours			24 hours			48 hours			72 hours			120 hours	
RvD1	2.7	±	1.4	3.8	±	2.4	3.5	±	2.9	2.5	±	1.3	1.4	±	0.7	2.5	±	1.4	3.4	±	2.3	2.9	±	1.8	2.9	±	1.5
RvD2		–			–			–			–			–			–			–			–			–	
RvD3		–			–			–			–			–			–			–			–			–	
RvD4		–			–			–			–			–			–			–			–			–	
RvD5		–			–			–			–			–			–			–			–			–	
RvD6		–			–			–			–			–			–			–			–			–	
AT-RvD1		–			–			–			–			–			–			–			–			–	
AT-RvD3		–			–			–			–			–			–			–			–			–	
PD1		–			–			–			–			–			–			–			–			–	
AT-PD1		–			–			–			–			–			–			–			–			–	
10S,17S-diHDHA		–			–			–			–			–			–			–			–			–	
22-OH-PD1		–			–			–			–			–			–			–			–			–	
Maresin 1		–			–			–			–			–			–			–			–			–	
7S,14S-diHDHA		–			–			–			–			–			–			–			–			–	
4S,14S-diHDHA		–			–			–			–			–			–			–			–			–	
RvE1	3.7	±	1.4	4.2	±	1.9	3.7	±	1.3	4.9	±	2.6	3.2	±	1.3	2.7	±	1.4	3.3	±	1.7	2.5	±	2.0	2.9	±	1.3
RvE2		–			–			–			–			–			–			–			–			–	
RvE3		–			–			–			–			–			–			–			–			–	
LXA_4		–			–			–			–			–			–			–			–			–	
LXB_4		–			–			–			–			–			–			–			–			–	
5S,15S-diHETE		–			–			–			–			–			–			–			–			–	
AT-LXA_4	0.2	±	0.2	0.1	±	0.1	0.1	±	0.1	0.1	±	0.1	0.1	±	0.1	0.2	±	0.2	0.1	±	0.1	0.2	±	0.2	0.3	±	0.3
AT-LXB_4		–			–			–			–			–			–			–			–			–	
LTB_4	17.6	±	5.0	13.5	±	3.2	22.7	±	10.7	21.1	±	10.3	13.9	±	5.7	26.1	±	3.3	18.9	±	6.4	15.4	±	4.1	18.2	±	10.7
20-OH-LTB_4	28.2	±	6.6	23.6	±	7.8	29.2	±	11.5	30.8	±	13.4	12.9	±	8.5	27.6	±	2.1	24.9	±	12.0	35.9	±	19.7	35.1	±	19.4
20-COOH-LTB_4		–			–			–			–			–			–			–			–			–	
5S,12S-diHETE		–			–			–			–			–			–			–			–			–	
PGD_2	10.6	±	5.5	8.7	±	3.7	19.1	±	8.1	21.1	±	3.6	9.7	±	5.3	7.4	±	2.9	6.1	±	3.9	11.7	±	7.0	6.2	±	3.2
PGE_2	49.6	±	20.2	32.5	±	15.6	54.9	±	19.1	60.7	±	23.9	14.5	±	8.2	27.5	±	11.1	16.1	±	5.6	41.7	±	18.3	19.6	±	8.9
PGF_{2a}	18.3	±	7.2	15.3	±	8.7	22.3	±	7.6	22.6	±	12.2	9.0	±	6.1	6.9	±	2.1	5.4	±	1.0	10.2	±	4.1	7.0	±	2.4
TXB_2	1431.9	±	750.3	925.3	±	334.7	1830.3	±	515.7	1428.7	±	590.6	285.8	±	152.0	483.8	±	261.7	352.4	±	177.2	813.7	±	527.4	500.4	±	303.2
17-HDHA	35.4	±	6.6	41.9	±	22.2	34.1	±	15.7	57.5	±	37.2	12.2	±	1.2	24.3	±	6.4	23.9	±	11.7	24.8	±	8.1	28.4	±	7.1
14-HDHA	279.0	±	105.9	243.9	±	161.2	421.9	±	248.5	568.0	±	405.2	31.8	±	11.0	179.7	±	83.6	99.8	±	72.1	282.8	±	131.5	121.8	±	69.3
7-HDHA		±			±			±			±			±			±			±			±			±	
4-HDHA	24.1	±	6.1	20.5	±	3.1	16.8	±	4.0	26.1	±	9.0	10.0	±	1.0	22.1	±	2.7	23.9	±	3.3	21.3	±	4.2	23.8	±	3.7
DHA	703971.5	±	86440.8	836481.0	±	104105.2	829277.3	±	294045.7	1139201.8	±	522820.9	583301.0	±	109001.4	770846.9	±	71472.6	1201747.4	±	380786.3	758734.1	±	18573.3	826464.5	±	47523.3
18-HEPE	354.4	±	138.6	214.7	±	42.1	186.5	±	31.5	202.3	±	108.5	96.6	±	10.3	213.0	±	67.1	200.5	±	62.9	211.0	±	39.3	257.6	±	58.9
15-HEPE	102.4	±	23.5	94.8	±	15.7	62.1	±	16.7	74.6	±	32.9	42.1	±	13.8	61.3	±	10.9	73.6	±	17.7	75.1	±	12.0	94.9	±	14.7
12-HEPE	1216.0	±	535.4	702.7	±	408.1	1070.6	±	312.6	1369.4	±	861.3	211.6	±	151.7	588.1	±	204.7	213.2	±	68.2	936.7	±	329.3	383.2	±	127.2
5-HEPE	42.7	±	4.0	45.0	±	12.1	26.3	±	5.3	38.6	±	20.3	13.1	±	1.5	35.0	±	10.9	44.3	±	13.8	39.3	±	13.3	41.8	±	10.7
EPA	57657.1	±	12761.9	48456.6	±	15814.1	44271.3	±	9937.4	63719.2	±	19730.3	27207.1	±	2357.5	64025.6	±	13490.2	82351.5	±	14796.4	71232.1	±	15507.6	70914.3	±	10940.3
15-HETE	753.7	±	332.4	622.9	±	186.8	1146.9	±	614.2	970.1	±	500.1	223.1	±	53.7	418.0	±	142.5	448.6	±	241.6	461.4	±	173.7	369.2	±	111.0
12-HETE	13213.1	±	7149.3	10859.0	±	4503.4	25080.4	±	13279.2	22123.4	±	12016.8	2666.2	±	1198.1	7154.5	±	3201.8	5639.0	±	4034.6	9053.8	±	4965.0	4830.3	±	3106.2
5-HETE	198.9	±	79.9	126.8	±	25.0	149.5	±	84.8	159.5	±	65.1	77.6	±	11.9	177.2	±	61.3	154.5	±	23.3	164.8	±	46.0	195.8	±	83.8
AA	176521.0	±	7403.9	204045.4	±	43253.1	264959.1	±	87952.2	275081.5	±	91856.3	164113.7	±	25175.7	185472.4	±	31728.0	280585.3	±	91690.6	186506.6	±	19360.7	191548.7	±	12979.1

Supplementary Table 3

Human serum LM-SPM profile from endotoxin challenge timecourse after n3 FA

Mediator		0 hour			1 hour			2 hours			4 hours			8 hours			24 hours			48 hours			72 hours			120 hours	
RvD1	100.1	±	95.5	8.5	±	2.4	16.4	±	14.2	20.4	±	18.1	11.8	±	5.8	32.9	±	28.6	12.2	±	5.8	10.7	±	7.4	9.8	±	6.4
RvD2		–			–			–			–			–			–			–			–			–	
RvD3		–			–			–			–			–			–			–			–			–	
RvD4		–			–			–			–			–			–			–			–			–	
RvD5		–			–			–			–			–			–			–			–			–	
RvD6		–			–			–			–			–			–			–			–			–	
AT-RvD1		–			–			–			–			–			–			–			–			–	
AT-RvD3		–			–			–			–			–			–			–			–			–	
PD1		–			–			–			–			–			–			–			–			–	
AT-PD1		–			–			–			–			–			–			–			–			–	
10S,17S-diHDHA		–			–			–			–			–			–			–			–			–	
22-OH-PD1		–			–			–			–			–			–			–			–			–	
Maresin 1		–			–			–			–			–			–			–			–			–	
7S,14S-diHDHA		–			–			–			–			–			–			–			–			–	
4S,14S-diHDHA		–			–			–			–			–			–			–			–			–	
RvE1	7.0	±	5.5	1.7	±	1.3	1.7	±	1.1	2.3	±	1.2	1.9	±	1.1	1.3	±	0.7	2.1	±	1.3	1.1	±	1.1	0.5	±	0.5
RvE2		–			–			–			–			–			–			–			–			–	
RvE3		–			–			–			–			–			–			–			–			–	
LXA_4		–			–			–			–			–			–			–			–			–	
LXB_4		–			–			–			–			–			–			–			–			–	
5S,15S-diHETE	241.5	±	240.5	51.3	±	46.6	14.0	±	12.2	19.2	±	17.7	58.2	±	57.7	86.8	±	85.2	29.1	±	28.4	18.1	±	16.9	46.6	±	46.1
AT-LXA_4	0.7	±	0.7	0.7	±	0.7	1.9	±	1.9	1.0	±	1.0	1.2	±	1.2	1.2	±	1.2	1.5	±	1.5	1.1	±	1.1	2.0	±	2.0
AT-LXB_4		–			–			–			–			–			–			–			–			–	
LTB_4	28.1	±	12.8	32.8	±	13.2	20.1	±	9.1	20.3	±	9.5	27.7	±	8.9	59.4	±	22.6	34.5	±	9.6	40.4	±	26.8	40.7	±	17.9
20-OH-LTB_4	53.3	±	31.0	67.1	±	28.4	34.1	±	27.2	26.6	±	14.4	19.1	±	4.2	54.7	±	25.4	37.5	±	11.1	29.1	±	21.6	41.5	±	18.1
20-COOH-LTB_4		–			–			–			–			–			–			–			–			–	
5S,12S-diHETE		–			–			–			–			–			–			–			–			–	
PGD_2	75.8	±	73.1	26.6	±	11.9	18.0	±	11.4	32.3	±	24.0	59.7	±	33.9	12.5	±	5.0	7.6	±	2.9	14.4	±	6.1	13.5	±	7.2
PGE_2	228.1	±	215.3	78.5	±	33.9	37.0	±	22.1	47.9	±	29.1	89.7	±	42.9	49.9	±	29.0	15.1	±	5.0	28.0	±	12.6	41.1	±	21.9
PGF_{2a}	156.2	±	145.7	33.0	±	22.6	23.0	±	11.0	28.4	±	13.6	40.7	±	15.1	14.1	±	4.7	10.0	±	5.5	17.0	±	7.4	12.7	±	4.7
TXB_2	1864.1	±	1767.6	860.2	±	408.1	500.9	±	285.3	696.4	±	456.6	845.7	±	394.2	473.1	±	250.4	230.2	±	183.3	167.4	±	95.9	378.0	±	202.1
17-HDHA	138.7	±	87.7	114.4	±	15.7	62.0	±	18.9	74.2	±	25.3	51.4	±	18.7	117.1	±	57.9	109.0	±	18.3	68.5	±	15.1	117.5	±	14.4
14-HDHA	1671.5	±	1617.4	684.5	±	361.5	322.6	±	267.2	446.5	±	360.5	381.9	±	279.3	955.5	±	774.6	106.9	±	21.8	303.9	±	144.2	484.8	±	301.8
7-HDHA		±			±			±			±			±			±			±			±			±	
4-HDHA	118.2	±	58.9	82.9	±	4.8	54.0	±	18.8	45.1	±	8.2	29.6	±	4.9	77.2	±	9.7	74.6	±	14.8	59.8	±	6.4	99.0	±	3.8
DHA	1398031.1	±	646486.9	1699337.3	±	542088.1	950249.8	±	597078.0	963500.4	±	318273.7	1003564.8	±	93523.1	1215914.1	±	602757.1	2685883.4	±	601168.0	1241295.1	±	716779.7	1371546.2	±	466156.2
18-HEPE	2117.0	±	1640.4	827.1	±	111.1	322.3	±	107.4	347.7	±	52.7	557.6	±	127.6	800.9	±	242.2	745.7	±	126.6	437.1	±	120.2	831.7	±	80.1
15-HEPE	547.9	±	374.3	263.9	±	38.9	142.9	±	73.6	192.2	±	118.2	151.2	±	40.7	286.8	±	81.7	236.8	±	39.7	175.6	±	77.2	316.7	±	18.2
12-HEPE	36875.7	±	36116.7	7207.7	±	3348.6	1155.3	±	402.2	1638.0	±	775.7	4370.8	±	2512.7	6661.7	±	5038.3	926.0	±	327.5	2182.5	±	1016.1	3763.9	±	2364.2
5-HEPE	162.5	±	33.4	145.0	±	16.7	72.5	±	16.2	86.9	±	29.4	47.3	±	7.2	331.9	±	136.4	187.8	±	13.8	135.2	±	19.1	190.6	±	21.9
EPA	292040.8	±	106836.7	235851.3	±	81807.0	154026.9	±	59948.5	113404.8	±	24275.8	126434.7	±	15831.4	276449.2	±	80125.5	408212.3	±	37006.1	184433.4	±	89974.0	288745.2	±	67887.5
15-HETE	1417.6	±	1142.8	1232.2	±	428.3	468.5	±	190.0	660.0	±	271.2	862.0	±	380.6	829.4	±	514.7	393.6	±	47.6	399.7	±	199.4	483.9	±	202.5
12-HETE	26891.6	±	25580.9	22161.9	±	10623.9	6164.3	±	3682.4	11783.6	±	9556.9	15837.3	±	10191.0	13196.5	±	10281.7	1873.1	±	810.2	5088.1	±	2492.1	7601.1	±	5203.9
5-HETE	217.5	±	100.7	153.8	±	51.2	102.7	±	39.4	100.7	±	35.1	94.4	±	22.5	229.2	±	56.9	243.9	±	68.3	164.9	±	67.2	210.0	±	92.9
AA	338750.0	±	154262.5	250300.9	±	127303.3	227189.8	±	145475.7	186389.9	±	86819.9	172658.2	±	64786.5	287169.7	±	95795.8	395088.9	±	19708.6	249912.6	±	140860.2	303038.5	±	85657.1

Supplementary Table 4

Human plasma LM-SPM profile from endotoxin challenge timecourse after placebo

Mediator		0 hour			1 hour			2 hours			4 hours			8 hours			24 hours			48 hours			72 hours			168 hours	
RvD1		–			–		0.1	±	0.1		–			–			–			–			–	
RvD2		–			–			–			–			–			–			–			–	
RvD3		–			–			–			–			–			–			–			–	
RvD4		–			–			–			–			–			–			–			–	
RvD5		–			–			–			–			–			–			–			–	
RvD6		–			–			–			–			–			–			–			–	
AT-RvD1		–			–			–			–			–			–			–			–	
AT-RvD3		–			–			–			–			–			–			–			–	
PD1		–			–			–			–			–			–			–			–	
AT-PD1		–			–			–			–			–			–			–			–	
10S,17S-diHDHA		–			–			–			–			–			–			–			–	
22-OH-PD1		–			–			–			–			–			–			–			–	
Maresin 1		–			–			–			–			–			–			–			–	
7S,14S-diHDHA		–			–			–			–			–			–			–			–	
4S,14S-diHDHA		–			–			–			–			–			–			–			–	
RvE1		–			–			–			–			–			–			–			–	
RvE2		–			–			–			–			–			–			–			–	
RvE3		–			–			–			–			–			–			–			–	
LXA$_4$		–			–			–			–			–			–			–			–	
LXB$_4$	4.2	±	1.9	3.0	±	1.5	4.9	±	2.4	4.8	±	2.3	2.1	±	1.4	2.5	±	1.5	2.6	±	1.9	2.5	±	1.6	2.6	±	1.9
5S,15S-diHETE	1.4	±	1.4	0.4	±	0.4	1.7	±	1.7	1.6	±	1.6	1.6	±	1.6	1.4	±	1.4	2.1	±	2.1	1.5	±	1.5	1.9	±	1.3
AT-LXA$_4$	2.1	±	1.9	1.6	±	1.6	2.1	±	2.1	1.8	±	1.7	1.7	±	1.7	0.7	±	0.6	0.2	±	0.1	0.7	±	0.7	0.2	±	0.2
AT-LXB$_4$		–			–			–			–			–			–			–			–	
LTB$_4$		–			–			–			–			–			–			–			–	
20-OH-LTB$_4$		–			–			–			–			–			–			–			–	
20-COOH-LTB$_4$		–			–			–			–			–			–			–			–	
5S,12S-diHETE		–			–			–			–			–			–			–			–	
PGD$_2$	0.1	±	0.1		–		0.1	±	0.1	0.1	±	0.1		–		0.1	±	0.1		–		0.1	±	0.1		–	
PGE$_2$		–			–			–			–			–		0.2	±	0.2	0.1	±	0.1		–			–	
PGF$_{2a}$	4.2	±	4.2	5.4	±	3.1	5.6	±	3.4	3.6	±	2.1	7.6	±	4.3	5.7	±	3.0	6.0	±	4.0	8.9	±	5.6	5.4	±	2.9
TXB$_2$	2.1	±	1.6	1.8	±	1.5	4.1	±	3.9	3.6	±	2.3	2.8	±	2.5	1.8	±	1.1	0.2	±	0.2	0.2	±	0.2		–	
17-HDHA	15.5	±	8.0	8.5	±	3.0	7.8	±	4.3	8.3	±	5.8	6.1	±	4.8	7.4	±	2.0	7.3	±	2.2	6.9	±	1.7	7.3	±	2.3
14-HDHA	7.6	±	3.0	2.4	±	0.9	2.3	±	0.9	1.4	±	0.8	1.0	±	0.4	3.8	±	1.6	4.0	±	0.6	3.6	±	0.6	2.5	±	1.0
7-HDHA	3.1	±	1.0	3.1	±	1.0	2.8	±	1.0	2.2	±	0.8	2.5	±	1.3	2.2	±	0.8	3.5	±	1.5	2.0	±	0.8	2.2	±	0.7
4-HDHA	4.7	±	1.1	-0.7	±	4.3	3.9	±	0.8	3.5	±	0.7	-2.3	±	4.6	5.2	±	1.7	4.8	±	1.6	3.6	±	1.4	2.9	±	1.4
DHA	17339.0	±	6990.5	11456.7	±	5557.5	11329.9	±	4141.1	12650.5	±	6850.2	9286.7	±	3061.8	15191.1	±	6635.8	20987.5	±	10674.8	14031.3	±	4661.7	15059.7	±	7436.2
18-HEPE	21.7	±	7.6	13.8	±	6.7	11.5	±	3.0	10.5	±	2.6	11.7	±	2.3	46.8	±	35.4	31.8	±	16.5	23.6	±	10.0	26.9	±	15.7
15-HEPE	18.9	±	1.9	10.4	±	4.9	10.2	±	1.2	6.7	±	0.4	8.2	±	1.9	14.9	±	6.6	10.4	±	3.3	7.4	±	1.7	6.4	±	1.0
12-HEPE	13.8	±	6.7	5.7	±	3.7	3.9	±	1.6	3.7	±	1.3	2.4	±	0.6	17.2	±	15.1	11.4	±	7.2	8.0	±	4.1	6.7	±	3.1
5-HEPE	6.1	±	1.6	3.0	±	1.1	2.3	±	0.5	3.0	±	0.8	1.5	±	0.5	7.2	±	6.6	4.3	±	2.8	4.0	±	3.3	3.6	±	2.2
EPA	4266.3	±	1740.3	2252.5	±	1490.1	1633.1	±	692.0	2391.2	±	1592.4	1173.4	±	552.4	7677.7	±	6678.5	7612.5	±	6586.0	4494.8	±	3416.4	4108.6	±	3102.7
15-HETE	31.2	±	18.0	20.4	±	12.8	15.5	±	6.8	15.8	±	6.4	8.9	±	2.7	13.9	±	9.4	14.3	±	7.0	16.0	±	10.6	14.8	±	7.6
12-HETE	27.6	±	16.4	15.2	±	9.0	14.7	±	6.0	13.8	±	4.2	8.9	±	2.6	15.9	±	9.7	15.7	±	7.5	19.4	±	10.7	13.7	±	6.7
5-HETE	14.0	±	4.6	12.1	±	7.4	8.5	±	3.2	8.7	±	4.1	6.6	±	3.1	9.0	±	5.4	9.5	±	5.4	9.6	±	4.1	8.3	±	4.6
AA	16396.2	±	6847.9	11376.0	±	6911.5	11635.1	±	5165.9	12270.7	±	7863.1	8309.9	±	3977.2	12130.4	±	5794.6	18476.4	±	10782.2	11836.6	±	4791.3	12353.1	±	6795.0

Supplementary Table 5

Human plasma LM-SPM profile from endotoxin challenge timecourse after n3 FA

Mediator	0 hour			1 hour			2 hours			4 hours			8 hours			24 hours			48 hours			72 hours			168 hours		
RvD1		–			–			–			–			–			–		0.1	±	0.1		–			–	
RvD2		–			–			–			–			–			–			–			–			–	
RvD3		–			–			–			–			–			–			–			–			–	
RvD4		–			–			–			–			–			–			–			–			–	
RvD5		–			–			–			–			–			–			–			–			–	
RvD6		–			–			–			–			–			–			–			–			–	
AT-RvD1		–			–			–			–			–			–			–			–			–	
AT-RvD3		–			–			–			–			–			–			–			–			–	
PD1		–			–			–			–			–			–			–			–			–	
AT-PD1		–			–			–			–			–			–			–			–			–	
10S,17S-diHDHA		–			–			–			–			–			–			–			–			–	
22-OH-PD1		–			–			–			–			–			–			–			–			–	
Maresin 1		–			–			–			–			–			–			–			–			–	
7S,14S-diHDHA		–			–			–			–			–			–			–			–			–	
4S,14S-diHDHA		–			–			–			–			–			–			–			–			–	
RvE1		–			–			–			–			–			–			–		3.5	±	3.5		–	
RvE2		–			–			–			–			–			–			–			–			–	
RvE3		–			–			–			–			–			–			–			–			–	
LXA_4	0.2	±	0.2	0.4	±	0.4	0.7	±	0.7		–		0.5	±	0.5	0.5	±	0.5	0.4	±	0.4	0.5	±	0.5	0.5	±	0.5
LXB_4	2.7	±	2.0	2.4	±	1.2	2.0	±	1.2	1.9	±	1.1	2.1	±	1.5	2.8	±	2.0	2.9	±	2.0	3.5	±	2.7	3.0	±	1.9
5S,15S-diHETE	1.9	±	1.2	2.6	±	1.8	2.6	±	1.7	2.8	±	1.4	2.6	±	1.7	3.1	±	2.1	2.8	±	1.7	2.2	±	1.3	2.6	±	1.6
AT-LXA_4	0.5	±	0.4	0.3	±	0.3	0.2	±	0.1	0.0	±	0.0	0.2	±	0.2	0.2	±	0.1	0.2	±	0.2	0.2	±	0.2	0.2	±	0.2
AT-LXB_4		–			–			–			–			–			–			–			–			–	
LTB_4		–		0.1	±	0.1	0.3	±	0.3	0.7	±	0.4	1.0	±	0.8	0.1	±	0.1		–			–		0.7	±	0.7
20-OH-LTB_4	0.5	±	0.5		–			–			–		0.2	±	0.2	0.3	±	0.3	0.4	±	0.4		–		0.7	±	0.7
20-COOH-LTB_4		–			–			–			–			–			–			–			–			–	
5S,12S-diHETE		–			–			–			–			–			–			–			–			–	
PGD_2		–			–		0.1	±	0.1	0.1	±	0.1		–			–			–			–			–	
PGE_2		–		0.1	±	0.1		–			–			–		0.1	±	0.1	0.1	±	0.1	0.1	±	0.1	0.1	±	0.1
PGF_{2a}	4.6	±	2.9	7.7	±	4.8	8.2	±	4.8	8.8	±	4.4	6.9	±	4.5	5.8	±	3.1	3.4	±	3.4	3.4	±	2.2	5.0	±	2.7
TXB_2		–			–		3.5	±	3.5		–			–			–		0.1	±	0.0		–			–	
17-HDHA	43.1	±	12.2	28.9	±	5.4	70.1	±	28.5	87.1	±	32.6	43.1	±	13.5	16.3	±	2.4	29.2	±	10.8	26.4	±	3.7	26.3	±	2.7
14-HDHA	12.4	±	5.9	6.5	±	2.2	11.3	±	4.7	20.0	±	8.0	7.9	±	2.5	6.7	±	1.6	10.2	±	2.6	11.0	±	0.9	11.4	±	2.7
7-HDHA	3.7	±	1.6	3.8	±	1.3	5.3	±	2.0	7.5	±	3.1	3.4	±	1.0	4.5	±	1.7	4.3	±	1.8	4.8	±	1.9	5.2	±	1.4
4-HDHA	9.3	±	3.5	8.1	±	3.6	8.8	±	4.7	13.1	±	8.5	7.1	±	2.8	5.8	±	2.3	8.2	±	4.2	7.1	±	3.1	8.3	±	3.2
DHA	23459.2	±	9777.1	19248.8	±	7652.1	25990.4	±	13947.4	22763.3	±	8690.0	22397.9	±	11012.2	16530.6	±	5339.7	19782.6	±	6302.5	23404.5	±	9363.4	28111.1	±	14932.0
18-HEPE	101.9	±	28.3	94.9	±	38.4	746.8	±	385.2	388.5	±	211.1	335.1	±	184.5	69.2	±	18.7	84.2	±	17.0	129.0	±	35.2	143.4	±	55.2
15-HEPE	60.5	±	12.5	40.2	±	13.7	47.5	±	7.4	94.3	±	32.0	50.4	±	20.3	22.2	±	6.2	28.4	±	3.6	27.3	±	5.0	41.1	±	14.1
12-HEPE	59.0	±	31.5	38.8	±	23.5	80.9	±	37.7	153.9	±	95.4	73.5	±	25.8	25.6	±	11.3	34.5	±	10.1	47.6	±	14.3	49.1	±	15.3
5-HEPE	22.0	±	10.3	17.6	±	12.0	31.0	±	20.5	37.9	±	19.6	12.3	±	4.2	7.6	±	4.6	19.4	±	9.9	15.1	±	8.8	13.1	±	6.1
EPA	18024.1	±	9129.0	14478.5	±	8822.4	17146.3	±	9962.6	22132.7	±	11499.7	15995.4	±	8780.4	11420.3	±	5634.5	14478.1	±	5439.6	17063.8	±	9443.4	15645.0	±	9747.7
15-HETE	27.3	±	14.5	22.3	±	13.5	23.2	±	13.3	23.4	±	11.9	12.0	±	4.6	13.6	±	8.3	20.5	±	12.1	13.6	±	7.3	19.4	±	12.8
12-HETE	19.0	±	10.3	16.0	±	8.9	30.2	±	13.9	19.5	±	11.6	12.3	±	3.9	12.4	±	6.3	14.9	±	7.9	12.6	±	5.4	14.9	±	7.6
5-HETE	13.2	±	4.5	9.8	±	5.0	9.4	±	4.4	10.9	±	6.6	5.6	±	2.6	5.4	±	2.9	11.7	±	7.4	6.8	±	3.6	9.3	±	5.2
AA	17424.7	±	7643.5	12257.3	±	5899.1	16776.9	±	10513.2	10836.8	±	5922.9	13551.0	±	8451.2	9826.7	±	3983.7	13956.2	±	5407.9	13422.4	±	6732.9	19160.9	±	13853.2
